# Advances in *in vitro* oocyte generation from pluripotent stem cells and ovarian stem cells

**DOI:** 10.3389/fendo.2025.1515253

**Published:** 2025-08-11

**Authors:** Mingxin Tian, Meixiang Zhang

**Affiliations:** ^1^ Center for Reproductive Medicine, First Affiliated Hospital of Zhengzhou University, Zhengzhou, China; ^2^ Henan Key Laboratory of Reproduction and Genetics, First Affiliated Hospital of Zhengzhou University, Zhengzhou, Henan, China

**Keywords:** primordial germ cell-like cells, ovarian stem cells, pluripotent stem cells, oogenesis, oocyte

## Abstract

Reproductive cells play a crucial role in transmitting genetic and epigenetic information from one generation to the next. Oocytes are fundamental to reproduction but human oocytes are difficult to obtain for clinical and research purposes because of ethical restrictions. However, *in vitro* induction systems have been established to differentiate pluripotent stem cells (PSCs) into human primordial germ cells (hPGCs). These induced hPGCs are referred to as hPGC-like cells. The discovery of ovarian stem cells (OSCs) also opened up a new avenue for studying the development of germline stem cells. In this review, we discuss the latest advances in the development of oocytes *in vivo* and *in vitro*, involving PSC-derived PGCs and ovary-isolated OSCs. Specifically, we focus on induction methods and differentiation mechanisms and discuss the associated technical challenges and future directions.

## 1 Introduction

Oocytes are the precursor cells of eggs in the female reproductive system. Mature oocytes are capable of being fertilized and developing into embryos and offspring (1, 2). The artificial generation of oocytes, which are usually derived from induced pluripotent stem cells (iPSCs) (3) or ovarian stem cells (OSCs) (4), provides significant opportunities for understanding the mechanism of oocyte development and for meeting clinical research needs. Human embryonic stem cells (hESCs) (5, 6), mouse embryonic stem cells (mESCs) (1, 2, 7), human iPSCs (hiPSCs) (6, 8, 9), and mouse iPSCs (miPSCs) (1, 2) can be induced to become primordial germ cell-like cells (PGCLCs), which can be combined with embryonic ovarian somatic cells to form reconstructed ovaries. Regardless of the cell type, the methods used to induce PGCLCs have been extensively developed. These methods commonly trigger the differentiation process towards inner cell mass and germ cells by incorporating factors, such as bone morphogenetic protein 4 (BMP4), leukemia inhibitory factor (LIF), epidermal growth factor (EGF), with or without stem cell factor (SCF) (5, 10, 11). However, the induction of PGCLCs to become oocytes typically needs to be carried out *in vivo*, for example, by injecting them into the ovaries, oviducts, or subcutaneous tissue of mice (5, 10, 11). Reconstructed ovaries can facilitate the maturation of mPGCLCs into oocytes, leading to the production of fertile offspring through *in vitro* maturation and fertilization, thereby providing the possibility of *in vitro* reproduction (1, 2). Therefore, pluripotent stem cells (PSCs) offer new possibilities for *in vitro* gametogenesis in humans and are also important models for investigating the mechanisms of PGC development.

The total number of follicles in mammals was believed to be established during the perinatal period, and that the production of ovarian oocytes ceased in adult women. However, mounting evidence in both mice (9, 12–14) and humans (4, 15, 16) indicate the presence of OSCs that have the ability to generate new oocytes. OSCs are typically isolated from mice or humans using antibodies against DEAD-box polypeptide 4 (DDX4) followed by magnetic-activated cell sorting (MACS) or fluorescence-activated cell sorting (FACS). Mouse OSCs (mOSCs) can generate fully functional oocytes *in vivo*, leading to the production of healthy embryos and offspring following fertilization. The transformation of OSCs to oocytes usually takes place within the *in vivo* environment; for example, by microinjection of mOSCs into the ovaries of recipient mice and allowing development to proceed (4, 14, 17, 18). In contrast, human OSCs (hOSCs) have been injected into human ovarian cortex tissue and then xenotransplanted into mice for development (4, 16, 19). Additionally, OSCs can be cultured *ex vivo* to successfully produce oocytes (15, 19, 20). These recent discoveries provide a fresh perspective for investigating the development of germline stem cells, and hold great promise for enhancing follicular reserve, preserving fertility, and addressing issues such as infertility and premature ovarian failure.

In this review, we discuss the latest understanding of cells that have the potential to develop into oocytes *in vivo* and *in vitro*, including PSC-induced primordial germ cells (PGCs) and ovary-isolated OSCs. Specifically, we focus on induction methods and differentiation mechanisms and discuss the associated technical challenges and future directions.

## 2 Normal oocyte development

The process of oogenesis begins during fetal life and continues until the end of the female’s reproductive period. During oogenesis, female gametes are produced from PGCs. In human, around 24th day of gestation, PGCs form in the wall of the yolk sac and migrate to the gonadal ridges around the 5th week of gestation. After reaching the primary gonad, cells repeatedly divide mitotically, forming oogonia. Then, meiosis occurs in the oocyte and stops in the bilinear phase of the pre-meiosis phase, which is called primary oocytes. At 20 weeks of gestation, the number of oocytes reaches its peak, with approximately 3.5 million in each ovary. During this period, oocytes enter meiosis, with the majority remaining arrested in the diplotene stage. However, 99.9% of oocytes fail to mature and undergo apoptosis at various stages of development (21, 22). Of these, approximately two-thirds undergo apoptosis between the pachytene stage of meiosis and the formation of the primordial follicle pool. At birth, there are approximately 2 million oogonia in the human female gonad. The majority of these follicles undergo atresia at various stages of follicular development, with only about 400 to 500 follicles capable of maturing and undergoing ovulation. An oocyte arrested at prophase I contains a large nucleus (also called an embryonic or germinal vesicle) with a visible nucleolus.

Folliculogenesis is a distinct sub-process that accompanies and supports oogenesis. Current research indicates that once a primordial follicle is activated, it embarks on a continuous and irreversible developmental trajectory, culminating in one of two outcomes: successful ovulation as a dominant follicle, or arrest at any stage following initiation, followed by atresia. The recruitment, activation, and growth of dormant ovarian follicles are initiated through the c-Kit/kit ligand-PI3K-PTEN-AKT signaling pathway. This pathway suppresses the activity of FOXO3 and mTOR, which are key regulators of follicle dormancy (23, 24). As a result, ovarian follicles progress from the primordial stage through primary and secondary stages, ultimately developing into Graafian follicles.

Prior to ovulation, the oocyte completes the first meiotic and becomes secondary oocytes, arresting at metaphase II (MII) of the second meiosis, waiting for fertilization. Reaching MII is essential for proper chromosome recombination during gamete fusion and zygote formation. The second meiotic division is completed only upon sperm entry into the oocyte.

## 3 Mechanisms of PGC specification

### 3.1 Signaling pathways involved in regulating hPGC specification

Our understanding of PGC induction mainly stems from studies in model mammals, including non-human primates, mice, and pigs. Accumulated evidence highlights differences in the cellular and molecular mechanisms governing PGC specification between mice and humans, particularly in terms of gene expression and signaling pathways. For instance, SOX17 is crucial for hPGC specification but not for mPGC induction, while *SOX2* is expressed in mPGCs but not in hPGCs. The induction system of hPGCLCs provides a valuable platform to investigate the mechanisms of specialization in hPGCs and hPGCLCs. Studies have shown that the expression of SOX17, TFAP2C, and BLIMP1 are essential for the specialization of hPGCLCs under the influence of BMP signaling.

**BMP4:** The BMPs belong to the transforming growth factor-beta superfamily and are crucial for embryo development. In the induction of PGCLCs, *BMP4* signaling through ALK2/3 is essential. In mice, *Bmp4* homozygous knockout embryos exhibit a lack of PGC development, highlighting the pivotal role of BMP4 in PGC induction *in vivo*. Furthermore, the response of epiblast cells to BMP is dose-dependent during the induction of PGCs (10).

**SOX17:** SOX17, a member of the SOX (SRY-related HMG box) family of transcription factors, was originally identified as a transcription factor for spermatogenesis. The discovery of SOX17 involvement in hPGC development began with the 4i culture of hPSCs and the *in vitro* differentiation of hPGCLCs. *In vitro* induction of hPGCLCs revealed SOX17 to be a key regulator of hPGC fate, while loss of SOX17 impairs hPGC specification. BLIMP1 works downstream of SOX17, which then represses endodermal and somatic genes (5). This pathway works only in hPGCs, and is not necessary for mPGC fate.

The transcription factor EOMES (T-box gene eomesodermin) functions upstream of SOX17 in hPGCLC specification. EOMES is upregulated in iMeLCs and activates SOX17 in response to WNT signaling. Initially expressed in the proximal epiblast before overt primitive streak formation, EOMES later becomes restricted to the primitive streak during gastrulation. While *EOMES* is expressed in iMeLCs, it is not expressed in PGCLCs or hPGCs. Knockout of EOMES impairs hPGCLC differentiation from hESCs and EOMES most likely acts downstream of WNT and TGFβ signaling pathways (11). This underscores EOMES being critical for human PGCLC specification in a cell-autonomous manner.

**BLIMP1:** BLIMP1, also known as PRDM1, encodes a zinc finger transcriptional repressor required for anterior endomesodermal cell fate and head induction. It can bind directly to and repress somatic cell proliferation genes. BLIMP1 expression is detected in human fetal gonocytes in the 12th week of gestation and is essential for hPGCLC specification. Mechanistically, BLIMP1 acts downstream of SOX17 to inhibit the developmental processes of neuron differentiation, gastrulation, and embryonic morphogenesis (10). Notably, BLIMP1 action is dose-dependent in hiPSCs. *BLIMP1^+/-^* hiPSCs exhibit a phenotype intermediate between that of wild-type and *Blimp1^-/-^* hiPSCs. This is consistent with the dose-dependent function of *Blimp1* observed in mice. As a complex and highly regulated developmental process, hPGC development involves the coordinated activity of numerous genes within a network. For instance, two other transcription factors, TFAP2C and PRDM14, play indispensable roles in hPGC specification.

**TFAP2C:** TFAP2C (also known as AP2-GAMMA) is a sequence-specific DNA-binding transcription factor involved in the activation of several developmental genes. Multiple studies have shown that TFAP2C is necessary for the formation of hPGCLCs. *TFAP2C^-/-^* hESCs are unable to induce the formation of hPGCLCs. However, when *TFAP2C^-/-^* hESC lines are injected into immune-deficient mice, they are capable of teratoma formation. This indicates that TFAP2C is not necessary for exit from primed pluripotency and somatic cell differentiation *per se*, instead it has a specific effect on the specification of hPGCLCs (25).

One human-specific role for TFAP2C in hPGCLCs involves the opening of naive-specific enhancers and the acquisition of naive-like pluripotency. In mice, the *Oct4* locus (also known as *Pou5f1*) is regulated by alternate enhancers. Specifically, the *Oct4* distal enhancer (DE) regulates *Oct4* expression in the inner cell mass and mPGCs, while the *Oct4* proximal enhancer (PE) regulates *Oct4* expression in the post-implantation epiblast of the mouse after implantation. During hPGCLC differentiation, the regulation of *OCT4* may involve the activation of DE and NE through TFAP2C binding. However, Chen et al. found that the lack of *OCT4* DE does not affect hESC differentiation into hPGCLCs, indicating that the DE is not a major regulator of hPGCLC specification (25). Recently, a naive enhancer (NE) that binds TFAP2C was discovered at the *OCT4* locus; however, no TFAP2C-binding NE exists in rodents. Aggregate differentiation without the NE results in a decreased percentage of hPGCLCs and reduced expression of *OCT4* RNA and diagnostic germ cell genes, such as *NANOS3*, *DND1*, *TFAP2C*, *SOX17*, and *PRDM1*. These data indicate that one of the mechanisms by which TFAP2C regulates human germ cell lineage formation is by opening naive-specific enhancers, with one enhancer corresponding to the NE at the *OCT4* locus (25).

TFAP2C may also partially regulate the expression of KLF4 during aggregate differentiation (25). In the absence of TFAP2C, the ground-state naive pluripotent transcription factor KLF4 is not expressed. Through detection of ATAC-seq signals and ChIP-qPCR with anti-TFAP2C antibodies, a new peak of open chromatin located 50 kb upstream of the *KLF4* locus was identified. TFAP2C binds to this region, which is referred to as the KLF4 element. Given that the NE at the human *OCT4* locus contains three AP2 sites and a KLF site, it is possible that regulation of the NE involves the combinatorial binding of both TFAP2C and possibly a KLF family member (25), although this remains to be determined.

In humans, TFAP2C plays a crucial role in the specification of germline cells, acting upstream of SOX17 through its binding to the *SOX17* promoter (26). However, the binding of TFAP2C at the *SOX17* locus alone is not sufficient to induce the dynamic upregulation of *SOX17* at the time of hPGCLC specification. However, there is coordinately enriched H3K27ac on both sides of the TFAP2C binding site in hPGCLCs, suggesting that this epigenetic regulation of TFAP2C may enable the expression of *SOX17* at the point of hPGCLC specification. It is worth noting that BMP signaling can also activate TFAP2C in a SOX17-independent manner, and both SOX17 and TFAP2C function upstream of BLIMP1 in the specification of human germ cells. Collectively, TFAP2C plays a critical role in the specification of PGCs.

**PRDM14:** PRDM14 is a member of the PRDI-BF1 and RIZ homology domain containing (PRDM) family of transcriptional regulators and is expressed in pre-implantation embryos and PGCs in mice and humans. However, in contrast to what is observed in mPGCs, during the specialization process of hPGCLCs, PRDM14 expression is delayed and significantly reduced in hPGCs (5). This suggests that PRDM14 may not be required for human PGC fate (11), or, alternatively, that low levels of PRDM14 are sufficient for hPGC development. Indeed, the loss of PRDM14 affects differentiation efficiency, leading to downregulation of hPGC marker genes, including *UTF1* and *NANOG*, while the re-expression of PRDM14 can rescue hPGCLC differentiation. In hESCs, knockdown of PRDM14 induces the expression of early differentiation marker genes and suppresses the expression of stem cell markers, whereas overexpression of PRDM14 significantly inhibits the expression of differentiation marker genes. These studies indicate a critical role of PRDM14 in hPGC fate by suppressing the expression of differentiation genes and maintaining hESC pluripotency.

A genome-wide RNA interference screen showed that PRDM14 binds to the proximal enhancer of pluripotency gene *OCT4* to regulate its expression in hESCs (27). Notably, PRDM14 regulates hPGC development probably through coordination with both TFAP2C and BLIMP1 because it shares a subset of transcription targets with TFAP2C and BLIMP1 (27), although the exact position of PRDM14 in the regulatory network of hPGC specification remains unknown.

### 3.2 Epigenetic reprogramming of hPGCs/hPGCLCs

Epigenetic reprogramming is another layer of regulation in hPGC development. Shortly after specification, throughout migration, and towards gonad colonization, epigenetic reprogramming takes place in hPGCs. Global genomic DNA demethylation at week 7 is a major epigenetic event during hPGC development. The inactivated X chromosome is reactivated in female hPGCs at 5.5–9 weeks, which is similar to that in mPGCs. The lowest level of hypomethylation occurs at week 10 for females and week 11 for males. The low levels of methylation are maintained until week 19, but global re-methylation starts in female PGCs at week 11 and in male PGCs at week 19.

hPGCLC-derived oogonia also display hallmarks of epigenetic reprogramming (3), such as genome-wide DNA demethylation, imprint erasure, and removal of aberrant DNA methylation in hPSCs, and acquire an immediate precursory state for meiotic recombination. Furthermore, the inactive X chromosome shows partial progressive demethylation and reactivation.

In the early embryonic development of mammals, there are two waves of DNA methylation reprogramming, one occurring shortly after fertilization, and the other in PGCs. Epigenetic reprogramming is believed to remove the epialleles acquired during previous stages of development, such as those generated during pre-fertilization gametogenesis and those formed by initial differentiation of the epiblast before gastrulation. Ground-state naive pluripotency in both mouse and human cells *in vitro* is associated with global DNA demethylation, and hPGCs *in vivo* are confirmed to be fully demethylated. The two stages of global DNA methylation reprogramming for attaining ground-state naive pluripotency may constitute crucial checkpoints in germ cell development. Intriguingly, in the hPGCLC model, the establishment of global DNA methylation reprogramming is not observed up to the fourth day of aggregate differentiation, yet the naive pluripotency marker, KLF4, is expressed in hPGCLCs. This suggests that the switch from a primed pluripotency state toward one that resembles the naive ground state in human germline cells precedes DNA methylation reprogramming. Another possibility is that once germline cells acquire transcriptome and chromatin states resembling ground-state naive pluripotency, they are protected from differentiation cues so as to maintain germline cell identity. This possibility is supported by the observation that human ground-state naive pluripotent stem cells do not readily respond to differentiation cues, leading to the formation of teratomas in immunocompromised mice, and necessitating re-priming to effectively differentiate into embryoid bodies (25).

## 4 Mechanisms of oocyte maturation

Oocyte maturation, the terminal phase of oogenesis, is essential for achieving fertilization competence and ensuring subsequent embryonic development. This process encompasses coordinated nuclear maturation (meiotic resumption), cytoplasmic maturation, and membrane maturation. The core regulatory mechanism involves the resumption of meiosis I, transitioning the oocyte from prophase I arrest to metaphase II readiness. Oocyte maturation is tightly regulated by a network of signaling pathways, with the LH surge serving as the primary physiological trigger. The LH surge coordinates maturation through three principal mechanisms: The LH surge coordinates maturation through three principal mechanisms: (1) resumption of meiosis I; (2) secretion of growth factors and hormones; and (3) calcium-mediated signaling.

**LH surge triggers meiotic resumption in oocytes.** The key intracellular signaling molecule that maintains meiotic block in oocytes is cAMP, whose levels are regulated by adenylate cyclases (ACs) and phosphodiesterases (PDEs). ACs are activated through G protein-coupled receptors such as GPR3, promoting the synthesis of cAMP, while PDEs break down cAMP. The inhibitory effect of cAMP is because cAMP can maintain the active state of PKA, thereby inhibiting the activity of MPF or degrading the subunits of MPF. The study found that the LH surge can restart meiosis of oocytes by interfering with the cAMP signaling pathway. However, cAMP in follicles has a dual effect of inhibiting and promoting nuclear maturation. When cAMP continues to rise in oocytes, its meiotic recovery is inhibited, while when cAMP is briefly increased, it promotes meiotic recovery in oocytes. The gap junction between granule cells and oocytes plays an important role in maintaining meiotic block. After the LH surge, the connection between cumulus cells and oocytes decreases, and the oocytes restart meiosis.

**LH surge promotes the secretion of growth factors** The LH surge increases the secretion of growth factors by acting on the LH receptors on follicle membrane cells and granule cells, thereby promoting the expansion of cumulus and the maturation of oocytes. EGF plays an important role in this process. EGF may cause Cx43 phosphorylation by activating the MAPK pathway, destroying the gap junction between granule cells and oocytes, thereby blocking the entry of meiotic inhibitors into the egg and promoting oocyte maturation.

**LH surge promotes hormone secretion** LH surges can also promote the secretion of a variety of hormones in granule cells, which are crucial to the maturation of oocytes. The LH surge promotes the synthesis of progesterone, steroid hormone and androgens, etc., can promote the maturation of oocytes. Follicular fluid meiosis activating sterol (FF-MAS) is a key factor secreted by granule cells to promote oocyte maturation. Studies have found that LH surge promotes the increase of FF-MAS. Recent research confirms that FF-MAS not only drives meiotic progression from MI to MII, but also stabilizes oocytes at the MII stage to facilitate fertilization.

**LH surge regulates calcium-mediated signaling** Calcium ions (Ca²^+^) play a key role in the maturation of oocyte nucleus. Nuclear maturation is dependent on calmodulin and Ca^2+^, which enhances the regulatory effect of cAMP by regulating the phosphodiesterase activity of oocytes. The release of Ca²^+^ intracellularly is one of the earliest signs of meiotic initiation. Under the action of the LH surge, phosphatidylinositol on the surface of the granule cell membrane hydrolyzes to generate Ca²^+^-released ligands, including diacylglycerol and inositol triphosphate (IP3). After IP3 binds to the receptor, Ca²^+^ of granules can be released and injected into the oocyte. After IP3 binds to the receptor, Ca²^+^of granules can be released from the cell and injected into the oocyte. IP3 can also enter the egg cell through the coupling pathway of gap junctions, stimulating the release of Ca²^+^in the oocyte; Ca²^+^may also enter the oocyte directly through gap junctions, and the eggs react to Ca²^+^induced Ca²^+^release.

## 5 *In vitro* oocyte induction from PSCs

PGCs are the foundational cells of the germline that are established during the early stages of embryonic development. PGCs play a crucial role in ensuring the generation of new organisms and are the source of germline totipotency (27). Animal models can provide valuable insights into human PGCs; however, the cellular and molecular mechanisms of PGC specialization observed in model animals often do not fully reflect the biology of human PGCs. Ethical considerations limit the study of human PGC development *in vivo* at early stages. However, *in vitro* induction systems have been developed to differentiate hESCs (5, 6) and iPSCs (3, 6, 8, 10) into human PGCs. These induced PGCs are known as human PGCLCs, and represent true *in vitro* counterparts of PGCs. Single-cell transcriptomics and cell lineage tracing have been used to elucidate the lineage trajectory and mechanisms of PGC specialization in the hPGCLC induction system. These studies indicate great promise for the future generation of functional human gametes from hPGCLCs *in vitro*. The progress made with hPGCLCs not only deepens our understanding of human reproduction but also provides a novel approach for treating infertility (**Table 1**).

**Table 1:** *In Vitro* PGCLC Induction from Human ESCs/iPSCs: Protocols and Efficiency.

Reference	Source	hPGCLC Induction Methods	Induction Condition	PGCLC Markers	PGCLC Induction Efficiency	Oocytes Generation	Data Availability	Key Finding
[3]	hiPSC	iMeLCs strategy	—	AGVT(+):TFAP2C-EGFP/ hVH-tdTomato	—	**the generation of xrOvaries:** xrOvaries were generated by aggregating d6 hPGCLCs with mouse fetal ovarian somatic cells of E12.5 embryos. **floating culture:** GK15,ROCK inhibitor **oogonia-like cells generation:** Transwell-COL membrane inserts,alpha-MEM; 10% FBS, β-ME, L-ascorbic acid, penicillin/streptomycin	GEO: GSE117101 DRA006618/DRA007077 (DDBJ)	• hPGCLCs differentiate progressively into oogonia-like cells during a long-term *in vitro* culture (approximately 4 months) in xrOvaries with mouse embryonic ovarian somatic cells. • The hPGCLC-derived oogonia display hallmarks of epigenetic reprogramming-genome-wide DNA demethylation, imprint erasure, and extinguishment of aberrant DNA methylation in hPSCs and acquire an immediate precursory state for meiotic recombination. • Partially inactive X chromosome shows a progressive demethylation and reactivation.
[5]	hESC	4i strategy	**hESC culture:** KODMEM; human LIF, bFGF, TGF-β1, CHIR99021,PD0325901, SB203580, SP600125 **hESC pre-induction:** (bFGF and TGFb) or Activin A **hPGCLC induction:** GMEM;BMP4/BMP2, human LIF, SCF, EGF	NANOS3^+^/TNAP^+^	Pre-induced embryoid bodies were differentiated into hPGCLCs until day 4–5, resulting in ~27% NANOS3/TNAP double-positive putative hPGCLC. hPGCLCs could be induced directly from 4i hESCs, exhibiting enhanced responsiveness and yielding ~45.5% hPGCLCs.	—	GEO: GSE60138.	• A defined model for hPGCLC specification from germline-competent hESCs. • Expression profiles of hPGCLCs match with authentic hPGCs. • SOX17 is the key regulator of hPGCLCs, which acts upstream of BLIMP1. BLIMP1 suppresses endoderm and other somatic genes during hPGCLCs specification. • CD38 glycoprotein is a cell-surface marker of the human germline.
[6]	hPSC	2D monolayer PGCLC differentiation	**hPSC culture**: mTeSR1 medium + 1% penicillin/streptomycin **Posterior epiblast induction(12h):** **primitive streak media:** aRB27 basal media;Activin, MCHIR99021, Y-27632 **PGCLC differentiation induction (3days)** all in aRB27 basal media; Day1: BMP4,XAV939, Y-27632 Day2: SCF, EGF, XAV939,Y-27632 Day3: BMP4, SCF, EGF, XAV939, and Y-27632	NANOS3-mCherry or: CXCR4^+^ PDGFRα^−^ GARP^−^	46.3 ± 8.5% (pure CXCR4 PDGFRα^+−^ GARP^−^ PGCLCs); 20-30%(NANOS3-mCherry PGCLCs)	—	GSE157475(scRNA-seq data); GSE210711 (bulk RNA-seq data)	• A simplified 2D platform to generate human PGCLCs within 3.5 days of hPSC differentiation. • Temporally dynamic WNT activation, followed by inhibition, increases efficiency of human PGCLC specification.Subsequent WNT inhibition promotes PGCLC specification and represses mesodermal genes. • hPSCderived PGCLCs can be easily purified by virtue of their CXCR4^+^PDGFRα^-^GARP^-^surface-marker profile. • Single-cell RNA-seq analysis shows that *in vitro*-derived PGCLCs have transcriptional similarities with *in vivo*-derived human fetal PGCs.
[8]	hiPSC	4i strategy	**complete hPGCLC maintenance medium (S-CM medium):** STO-CM; β-ME, L-ascorbic acid, recombinant human SCF **hPGCLC basal medium:** Glasgow’s MEM; KSR, NEAA, sodium pyruvate, glutamine,and penicillin-streptomycin **STO-CM:** mitomycin Ctreated STO cells was cultured in hPGCLC basal medium for 24 h, removing cells by centrifugation, and storing frozen at 20°C until use.	CD38(+)	30%-40%	—	GSE174485	• A novel system perpetually expands hPGCLCs. • HPGCLCs expand as a homogeneous cell population maintaining the germline features. • Long-term culture (LTC) hPGCLCs can be converted to embryonic germ cell-like cells. • The CpG methylation of LTR5_Hs is specifically decreased in LTC-hPGCLCs.
[10]	hiPSC	iMeLCs strategy	**hiPSC culture:** DMEM/F12; bFGF **iMeLCs induction:** GMEM; ACTA, CHIR (or WNT3A), **hPGCLC induction:** GMEM; LIF, BMP4/BMP2/ BMP7/BMP8A, SCF, EGF,	BTAG(+): BLIMP12A-tdTomato/ TFAP2C-2A-EGFP	hPGCLCs could be induced directly from hiPSCs; by day 6–8, a distinct population of BTAG(+) cells (~20%) appeared. Pre-induced iMeLCs were differentiated into hPGCLCs, resulting in ~30–40% BTAG(+) cells at day 2 and up to ~60% at day 4.	—	GEO: GSE67259	• Robust induction of hPGCLCs from primed hiPSCs occurs via incipient mesoderm-like cells • EpCAM and INTEGRINa6 are identified as markers for hPGCLC purification. • hPGCLCs avoid activation of a somatic program and undergo epigenetic reprogramming • BLIMP1 stabilizes germline transcription and represses neuronal differentiation.
[11]	hESC	iMeLCs strategy	**iMeLCs induction:** GMEM; CHIR99021, activin A, **hPGCLC induction:** GMEM; human LIF, BMP4, EGF	TNAP/cKIT(+) ITGA6/EPCAM(+)	UCLA6 (46XY) showed the highest germline potential, yielding ~35% PGCLCs by day 4, whereas UCLA9 (46XX) had the lowest, averaging <1%	—	—	• The transcription factor EOMES is required for efficient induction of human PGCLCs from hESCs. • Primitive streak gene expression is also associated with germline competency of hESCs, and PGCLC competency can be attributed to the appropriate induction of T and EOMES downstream of TGFβ and WNT signaling. • ITGA6 and EPCAM can be used to isolate human PGCs from embryonic ovaries.
[11]	hESC	4i strategy	**4i hESC culture:** KODMEM; human LIF, bFGF, TGF-β1, CHIR99021, PD0325901, SB203580, SP600125 **hPGCLC induction:** GMEM; BMP4 or BMP2, human LIF, SCF, EGF					
[25]	hESC	iMeLCs strategy	**hESC culture:** DMEM/F12; on MEFs, human FGF basic **iMeLCs induction:** GMEM; CHIR99021, activin A **hPGCLC induction:** GMEM; human LIF, BMP4, EGF	ITGA6/EPCAM	—	—	GEO: GSE120648 GEO: GSE93126.	• The chromatin and transcriptome of hPGCs resembles ground-state naive hESCs. • The TFAP2C-regulated OCT4 naive enhancer is involved in hPGC formation. • TFAP2C is required for hPGC formation and expression of KLF4.
[26]	hESC	iMeLCs strategy	**hESC culture:** DMEM/F12; on MEFs, human FGF basic **iMeLCs induction:** GMEM; CHIR99021, activin A, **hPGCLC induction:** GMEM; human LIF, BMP4, EGF	NANOG/SOX17/TFAP2C (N/S/T)	—	—	GEO: GSE140021	• Human germline cell specification begins from a transitional pluripotent state. • Human primordial germ cells are specified from lineage-primed progenitors. • Lineage-primed TFAP2A progenitors have gastrulating and amnion cell identity. • TFAP2C regulates SOX17 at the point of human primordial germ cell specification.
[28]	hiPSC	iMeLCs strategy	**iMeLCs induction:** GMEM; CHIR99021, activin A **hPGCLC induction:** GMEM; BMP4 ,SCF, LIF, EGF, **hPGCLC expansion culture:** m220 feeders, DMEM; KSR, FBS,SCF,forskolin/SCF/bFGF	BTAG(+): BLIMP1-tdTomato /TFAP2C-EGFP	—	**the generation of xrOvaries:** xrOvaries were generated by aggregating of d6 hPGCLCs or d6c30 hPGCLCs with mouse embryonic ovarian somatic cells of the ICR strain at E12.5. **floating culture:** GK15,ROCK inhibitor **xrOvary culture:** Transwell-COL membrane inserts, xrOvary culture medium[alpha-MEM; FBS, β-ME, L-ascorbic acid, penicillin/streptomycin	GEO:GSE147498 (RNA-seq data); GEO:GSE147499 (whole-genome bisulfite sequence data); GEO:GSE148415 (aCGH data).	• Induced pluripotent stem cell‐derived hPGCLCs expand ~10^6^‐fold under forskolin/SCF/bFGF *in vitro* over a period of four months. • During expansion, hPGCLCs maintain an early hPGC(LC) transcriptome and DNA methylome. • hPGC(LC) specification and epigenetic reprogramming are genetically dissociable. • Expanded hPGCLCs differentiate into oogonia with epigenetic reprogramming in xenogeneic reconstituted ovaries.
[29]	hESC	iMeLCs strategy	**iMeLCs induction:** GMEM; CHIR99021, activin A **hPGCLC induction:** GMEM; human LIF, BMP4, EGF, SCF	SOX17/NANOS3	—	—	GSM5808297 (FGFR3-sorted cells); GSM5808298 (UCLA1 day 4 aggregate)	• Human PGCs express FGFR3. • FGFR3 RNA and protein are repressed as PGCs enter prophase I of meiosis I. • FACS using FGFR3 antibodies can be used to enrich for PGCs from prenatal ovaries. • PGCLCs express FGFR3 following differentiation from hESCs.

BMP4, bone morphogenetic protein 4; β-ME, β-mercaptoethanol; EGF, epidermal growth factor; ESCs, embryonic stem cells; iMeLCs, incipient mesoderm-like cells; iPSCs, induced pluripotent stem cells; LIF, leukemia inhibitory factor; NEAA, non-essential amino acids; PGCs, primordial germ cells; PGCLCs, primordial germ cell-like cells; PSC, pluripotent stem cells; SCF, stem cell factor; xrOvaries, xenogeneic reconstituted ovaries.

aRB27 basal media: Advanced RPMI 1640 medium,1% B27 supplement, 0.1mMNEAA, 100 U/mL Penicillin ,0.1 mg/mL Streptomycin, and 2mM L-glutamine;

GK15 medium: GMEM; 15% KSR, 0.1 mM NEAA, 2 mM L-glutamine, 1 mM sodium pyruvate, and 0.1 mM β-ME.

### 5.1 *In vitro* induction and expansion of PGCLCs

#### 5.1.1 Methodologies for inducing mPGCLCs

**Figure 1:**
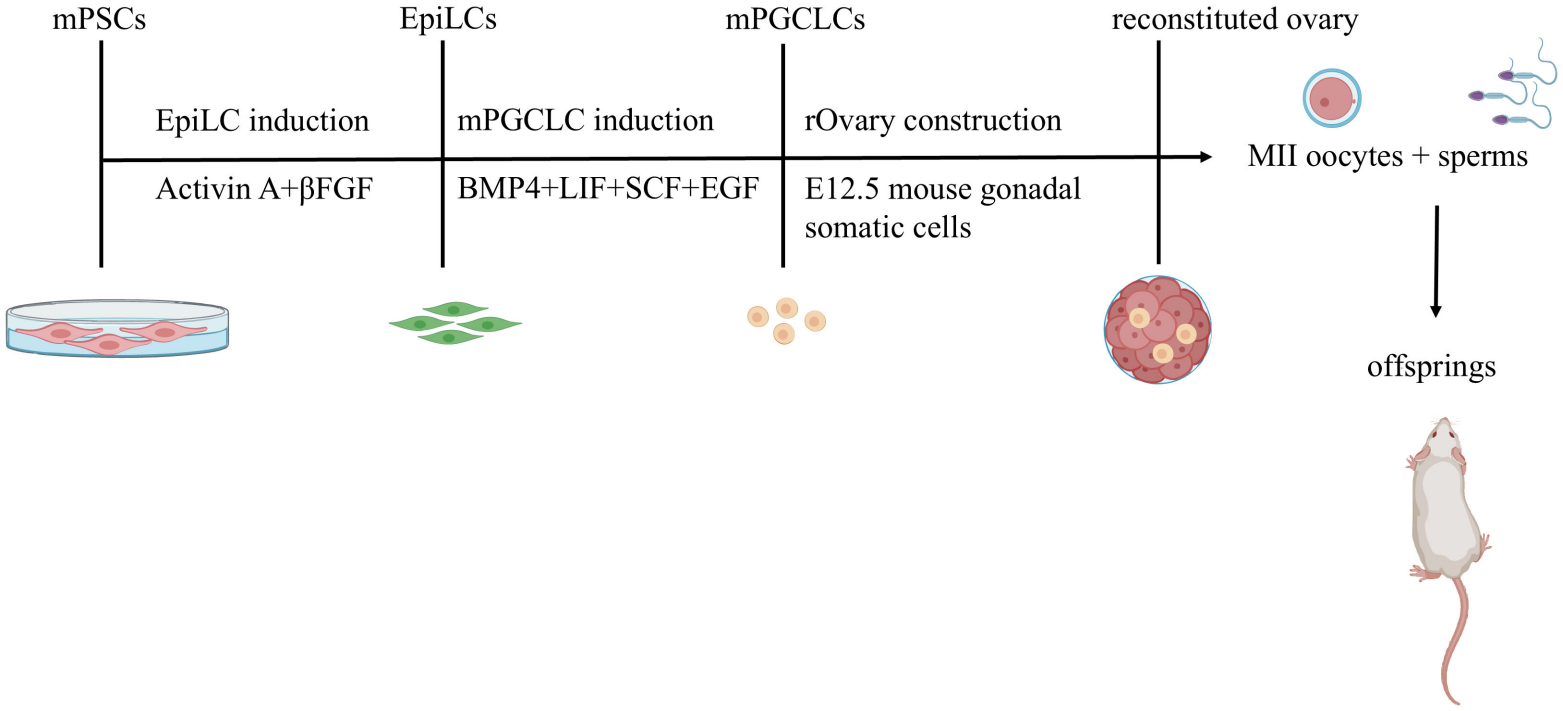
*In Vitro* Reconstitution of Functional Oocytes from Mouse Pluripotent Stem Cells with Live Offspring Generation. Mouse embryonic stem cells (mESCs) and induced pluripotent stem cells (iPSCs) are differentiated into epiblast-like cells (EpiLCs) using activin A and β FGF. EpiLCs subsequently undergo primordial germ cell-like cell (mPGCLC) specification via BMP4, LIF, SCF, and EGF stimulation during migration. mPGCLCs co-cultured with mouse embryonic ovarian somatic cells self-organize into reconstituted ovaries(rOvaries). These structures support in vitro maturation or *in vivo* transplantation (ovarian/subcutaneous sites) to generate fertile oocytes.

Mouse oocytes can be induced from mouse PSCs (1, 2, 7). First, mESCs are stimulated with activin and fibroblast growth factor (FGF) to induce them to become epiblast-like cells (EpiLCs). Then, under the influence of BMP4, LIF, SCF, and EGF, mEpiLCs differentiate into mPGCLCs during the migration stage. The characteristics of these mPGCLCs are similar to those of E9.5 mPGCs. Essentially the same protocol can be used to induce oocytes from miPSCs (**Figure 1**).

#### 5.1.2 Methodologies for inducing hPGCLCs

In a similar way, hPGCLCs can be derived from hESCs and hiPSCs. There are two main strategies for inducing hPGCLCs: the incipient mesoderm-like cell (iMeLC) strategy (3, 10, 25, 26, 28, 29) and the 4i strategy (5, 11).

**Figure 2:**
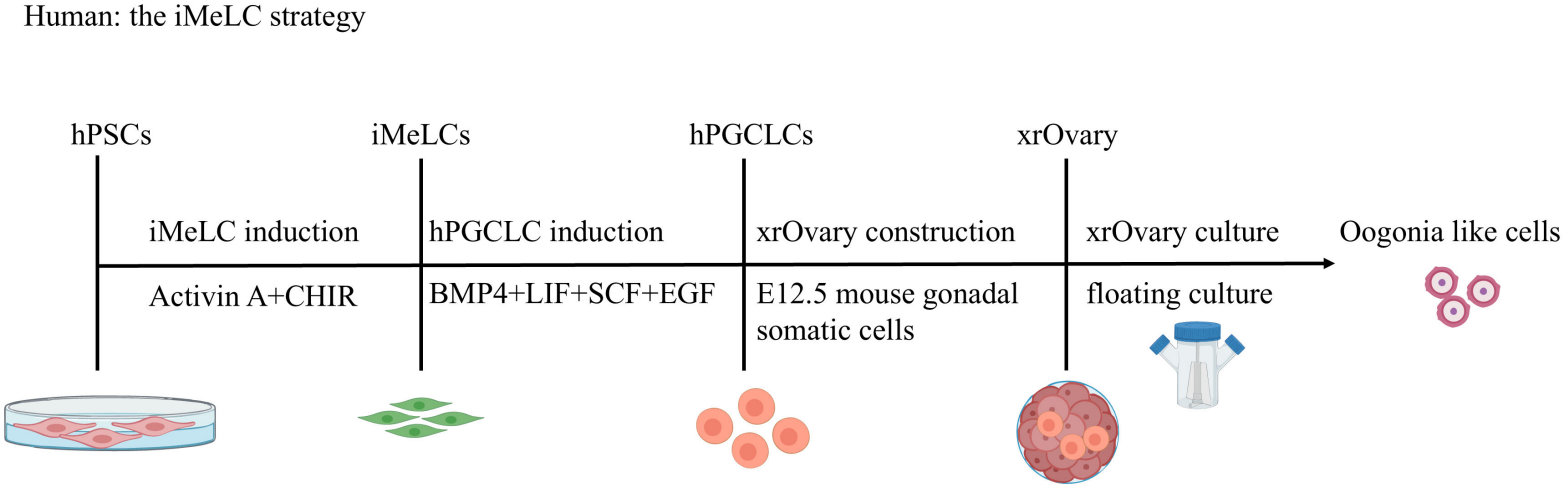
Stepwise Induction of hPGCLCs via iMeLC and Specification of Oogonia-like Cells. The iMeLC strategy is a two-phase induction protocol. Phase 1: activin A and CHIR-driven differentiation of human pluripotent stem cells (hPSCs) to incipient mesoderm-like cells (iMeLCs). Phase 2: BMP4/SCF/LIF/EGF-mediated induction of human primordial germ cell-like cells (hPGCLCs) from iMeLCs. hPGCLCs subsequently differentiate into oogonia-like cells in xenogeneic ovarian reconstitution cultures with mouse embryonic ovarian somatic cells.

**The iMeLC strategy** (**Figure 2**). This strategy is a two-phase induction process from hPSCs to iMeLCs and then to hPGCLCs (27). First, hPSCs are cultured under conventional conditions. During the first phase, hPSCs are induced with activin A (ACTA) and CHIR (a WNT signaling agonist) to form incipient mesoderm-like cells (iMeLCs),which exhibit a similar state to EpiLCs induced from mouse ESCs/iPSCs. In the second stage from iMeLCs to hPGCLCs, iMeLCs are cultured in medium supplemented with BMP4, SCF, LIF, and EGF to induce their differentiation into hPGCLCs.

The duration of ACTA and CHIR stimulation is critical for hPSCs to acquire a capacitated iMeLC state. Longer stimulation results in excessive upregulation of mesodermal/endodermal properties and depletes the capacity for BTAG(+) cell induction (10). Both ACTA and CHIR/WNT3A are essential; however, both BMP4 and bFGF are detrimental to iMeLC induction. Notably, inhibition of FGF receptor (FGFR) signaling by a specific inhibitor (FGFRi, PD173074) during iMeLC induction leads to more robust proliferation/survival of cells in the aggregates (10).

**Figure 3:**
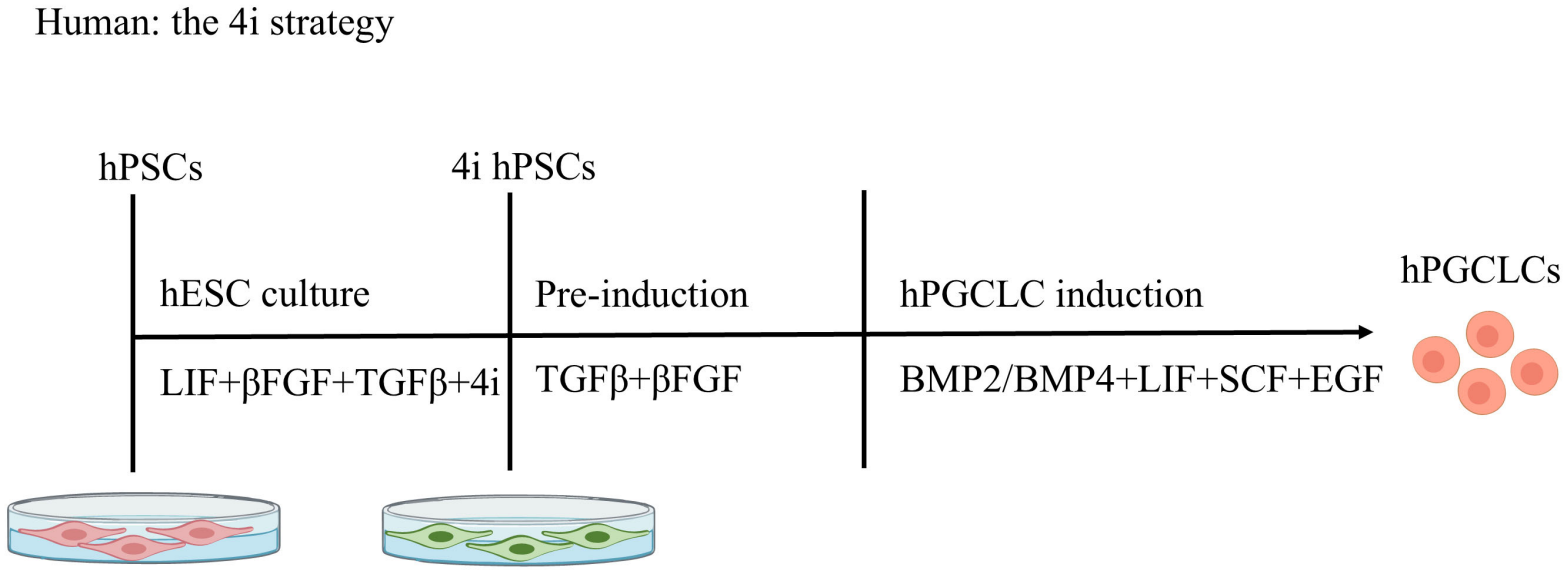
Stepwise Induction of hPGCLCs: The 4i strategy. Human pluripotent stem cells (hPSCs) are cultured in medium containing four inhibitors ("4i") and primed with TGF-β and bFGF. These pre-induced cells are then differentiated into primordial germ cell-like cells (hPGCLCs) using BMP2/BMP4, LIF, SCF, and EGF. The 4i comprises: CHIR99021 (GSK-3 inhibitor); PD0325901 (MEK inhibitor); SB203580 (p38 MAPK inhibitor); SP600125 (JNK inhibitor).

**The 4i strategy** (**Figure 3**). The 4i (four inhibitor) strategy refers to the use of four inhibitors. These are CHIR99021, a GSK-3 inhibitor; PD0325901, a MEK inhibitor; SB203580, a p38 MAPK inhibitor; and SP600125, a JNK inhibitor. hESCs are cultured in a medium containing these four inhibitors and pre-induced with TGFβ and bFGF. These pre-induced cells are then further guided towards hPGCLCs by culture in Glasgow’s minimum essential medium (GMEM) supplemented with BMP2/BMP4, LIF, SCF, and EGF. 4i culture is a key step that renders cells competent to acquire the hPGC fate (10). In the pre-induction step, activin A can be used as a substitute for TGFβ and bFGF (5).For hPGCLC induction, BMP4 signaling through activin receptor-like kinase 2/3 (ALK2/3) is essential. BMP2, but not BMP7 or BMP8A, can replace the role of BMP4 at an essentially identical concentration (10).

Notably, Sasaki et al. pointed out that hPSCs produced by the 4i strategy are not in the postulated naive state, but are in a type of peri-gastrulating epiblast-like state, similar to iMeLCs (10); mesodermal markers rather than genes for naive pluripotency are upregulated in these cells. Therefore, primed hESCs or 4i-cultured hESCs consistently generate hPGCLCs from iMeLCs or mesendoderm precursors (pre-MEs) (25). Meanwhile, the starting culture conditions (4i on mouse embryonic fibroblasts versus primed media on mouse embryonic fibroblasts) ultimately yields PGCLCs with similar transcriptional identities (11). RNA-seq analysis showed that PGCLCs generated from either primed media- or 4i-cultured hESCs clustered together, and were distinct from the undifferentiated hESCs. This means that hESC-derived PGCLCs generated through the iMeLC or 4i strategy exhibit similar molecular profiles.

Accordingly, recent studies have demonstrated the *in vitro* specification and reconstitution of the mouse germline by PSCs; mESCs/iPSCs with ground state pluripotency were induced into pre-gastrulation epiblast-like cells, which were in turn induced into PGCLCs with robust capacity for oogenesis and the generation of offspring. These findings indicate a conceptual framework and feasibility for the reconstitution of human germ cell development *in vitro* (10).

#### 5.1.3 Varied PGCLC induction efficiency among PSC types

Different PSCs exhibit distinct potentials for PGCLC generation. Previous studies have systematically characterized the PGCLC-inducing capacities across various PSC types, including mESCs, mouse epiblast stem cells (EpiSCs), naïve hPSCs, and primed hPSCs. mEpiSCs retain characteristics of the original epiblast cells and serve as a potential source for generating germ cell-like cells *in vitro*. Under self-renewing conditions, cells positive for STELLA (also known as PGC7/DPPA3), a marker of established PGCs, can be derived from mEpiSCs. However, the emergence of these positive cells occurs at a low frequency (1.5%) even in the presence of BMP4, and the functional relevance of these cells *in vivo* remains uncharacterized (30). In contrast, EpiLCs derived from naive mESCs demonstrate significant germline potential. When cultured with BMP4, the induction efficiency of PGCLCs can reach up to 40% (30).

The efficiency of generating hPGCLCs *in vitro* varies significantly depending on the stem cell type and induction strategy. Conventional hESCs spontaneously differentiate into hPGCLCs at a low efficiency of approximately 5% without directed induction (5). In contrast, direct induction of naive hiPSCs yields 15% BTAG^+^cells by day 4 (D4) (10). When hESCs are treated with a 4i culture system, the proportion of cells co-expressing TNAP and NANOS3 increases to 45.5% (5). The most efficient approach, the iMeLC strategy, achieves up to 60% BTAG^+^ cells by D4 (10). Furthermore, Zhu (31) utilized a novel type of formative PSCs (fPSCs) derived from human extended pluripotent stem cells (hEPSCs) to induce PGCLCs. These fPSCs, termed AF9-hPSCs, exhibit intermediate pluripotency features and demonstrate transcriptomic similarity to human E8-E9 epiblast cells. Under BMP4 treatment, the PGCLC induction efficiency reached 26.6% by day 4 (31).

The previously mentioned hPGCLCs derived from early progenitor cells were induced as three-dimensional aggregates in the presence of growth factors, including BMP4,usually using high BMP4 concentrations (200–500 ng/mL). In contrast, Vijayakumar (6) reports a simplified two-dimensional monolayer culture system that generates consistent and reproducible NANOS3-mCherry^+^ hPGCLCs with 20–30% purity within 3.5 days of *in vitro* differentiation, even at a 25-fold lower BMP4 concentration.

As mentioned earlier, 4i-cultured hPSCs reside in a peri-gastrulation epiblast-like state, similar to iMeLCs (10). hESCs/iPSCs have differentiation potential and other properties distinct from mESCs/iPSCs and bear a primed pluripotency with similarity to mouse EpiSCs, which resemble post-gastrulation epiblasts (10). The robust induction of hPGCLCs from hiPSCs in a primed pluripotent state, particularly through iMeLCs, is therefore surprising because EpiSCs with primed pluripotency exhibit little, if any, competence to achieve a germ cell fate (10). Further investigation indicated that hiPSCs bear properties intermediate between those of EpiLCs and EpiSCs (10). Chen et al. recently showed that iMeLCs in the lineage trajectory represent a transitional pluripotent state, exhibiting characteristics of both naive and primed hESCs. This state is known as the “germinal pluripotent state” (26).

#### 5.1.4 *In vitro* expansion of PGCLCs

The difficulty in *in vitro* expansion of PGCs has been a major obstacle in advancing PGC biology research. Studies have shown that chemical agents (7, 28) including selective inhibitors for PDE4 (e.g., Rolipram), agonists for retinoic acid (RA) signaling, and Forskolin, combined with cytokines (10, 28) such as SCF, LIF, and EGF, exert additive effects on the proliferation of PGCLCs. Ohta et al. successfully established a method for the *in vitro* expansion of mPGCLCs by stimulating the intracellular production of cyclic AMPs (cAMPs) with forskolin and rolipram (7). Their study demonstrated that mPGCLCs could proliferate for over a week *in vitro*, expanding up to ~50-fold. Subsequently, Yusuke Murase explored the *in vitro* expansion conditions for hPGCLCs (28). Their results showed that hPGCLCs can be propagated by a magnitude of at least ~1×10^6^ fold (~20 doublings) during a period of ~4 months with the maintenance of early hPGC properties in the presence of SCF, bFGF, and forskolin. The significant differences in expansion levels and culture durations between mPGCLCs and hPGCLCs may reflect fundamental differences in the intrinsic properties of human and mouse embryonic germ cells: human embryonic germ cells reach 7,000,000 in females (14–19 weeks) and 2,000,000 in males (19 weeks), whereas mouse embryos develop only 25,000 germ cells in both sexes by E13.5 (28). This discrepancy may be due to the unique long-term proliferation mechanisms of human cells or the intrinsic regulatory differences that limit the cell mitotic cycle in mouse cells.

#### 5.1.5 Markers of hPGCs/hPGCLCs

Multiple proteins located on the cell membrane of human PGCs have been used to enrich PGCs from single-cell suspensions of human embryonic and fetal tissues, as well as from *in vitro* PGCLCs. These include cKIT, TNAP, PDPN, CD38, ITGA6 (also known as Integrinα6), and EPCAM. Combinations of multiple markers can improve isolation accuracy. Combinations, such as NANOS3/NANOG/SOX17, NANOS3/TNAP, and OCT4/TFAP2C have been used as markers for hPGCLCs, and NANOG/SOX17/TFAP2C (N/S/T) as markers for hPGCs. Additionally, EpCAM and Integrinα6 are markers for hPGCLC purification. hPGCLCs can be isolated *in vitro* (10) and from embryonic ovaries (11) using EPCAM and Integrinα6.

hPGCs express fibroblast growth factor receptor 3 (FGFR3) (29), but this expression is downregulated as PGCs progress into prophase I of meiosis I. FACS using FGFR3 antibodies can be used to enrich for PGCs from prenatal ovaries. FGFR3 can therefore serve as a diagnostic surface marker to improve PGCLC differentiation protocols.

### 5.2 mPGCLC-derived oocytes and generation of offspring

As mentioned earlier, the method to induce mouse PSCs into mPGCLCs has been extensively developed. mPGCLCs can generate fully functional oocytes *in vitro*. When co-cultured with mouse embryonic ovarian somatic cells, aggregates of mPGCLCs and ovarian somatic cells are often referred to as reconstructed ovaries. Reconstructed ovaries can induce oocyte production either through complete *in vitro* culture or re-implantation into mouse ovaries or subcutaneous tissue (1, 2). The entire process is divided into three stages: *in vitro* differentiation, *in vitro* growth, and *in vitro* maturation. During *in vitro* differentiation, mPGCLCs differentiate into primary oocytes at the secondary follicle stage, and during the *in vitro* growth stage, they further differentiate into mature oocytes. MII oocytes derived from both mESCs and miPSCs can be fertilized to produce offspring, demonstrating successful *in vitro* oogenesis.

However, during development, approximately half of PGCLC-derived oocytes/zygotes fail to extrude the second polar body. After *in vitro* fertilization, this results in the digynic triploid (MMP) phenotype or the digynic diploid (MM) phenotype with failed fertilization. This defect contributes to a low birth rate from the 2-cell embryos derived from PGCLCs. Further investigations of the underlying mechanisms of this failure are needed (1). These advances in mice serve as a basis for *in vitro* reconstitution of human germ cell development.

### 5.3 hPGCLC-derived early oocytes

hPGCLCs progressively differentiate into oogonia-like cells during long-term *in vitro* culture in xenogeneic reconstituted ovaries (xrOvary) with mouse embryonic ovarian somatic cells (3, 28). In this system, hPGCLCs are first aggregated with mouse embryonic ovarian somatic cells at E12.5 and then cultured under floating conditions in GK15 (GMEM+15% KSR) containing Y-27632 in a U-bottom 96-well plate for 2 days to form xrOvaries. Finally, xrOvaries are transferred to Transwell-COL membrane inserts and cultured in minimum essential medium-alpha (MEM-α) containing fetal bovine serum (FBS), 2-mercaptoethanol, L-ascorbic acid, and penicillin/streptomycin, under liquid–gas interface conditions.

qPCR or RNA-seq analysis of c30ag7,c30ag35, and c30ag77 cells (The term 'c30ag7' means hPGCLCs cultured *in vitro* for 30 days [c30], followed by 7 days of aggregation culture with mouse embryonic ovarian somatic cells [ag7].The same naming rule applies to subsequent terms), indicates that marker genes for oogonia/gonocytes, including *DPPA3*, *PRAME*, *PIWIL2*, *DAZL*, and *DDX4*, were more rapidly up-regulated compared with in d6 hPGCLC-derived cells (28). Whole-genome bisulfite sequence analysis revealed the genome-wide DNA methylation 5-methylcytosine (5mC) profiles of hPGCLC-derived cells in xrOvaries. The genome-wide 5mC levels in hPGCLC-derived c30ag77 cells reach approximately 15% (28), a value equivalent to that of human oogonia/gonocytes after 7–10 weeks of development and of ag120 cells (approximately 13%) (3, 28). Unsupervised hierarchical clustering, principle component analysis, and the expression profiles of a gene set that characterizes developmental progression from hPGCLCs to oogonia/gonocytes provided a consistent outcome: c30ag35 and c30ag77 cells represent early- and late-stage oogonia/gonocyte-like cells, respectively (28). The above results from both studies collectively indicate that ag120 and c30ag77 cells from long-term cultures are likely oocytes. These two studies offer fresh insights into human *in vitro* oogenesis.

## 6 *In vitro* oocyte induction from OSCs

**Figure 4:**
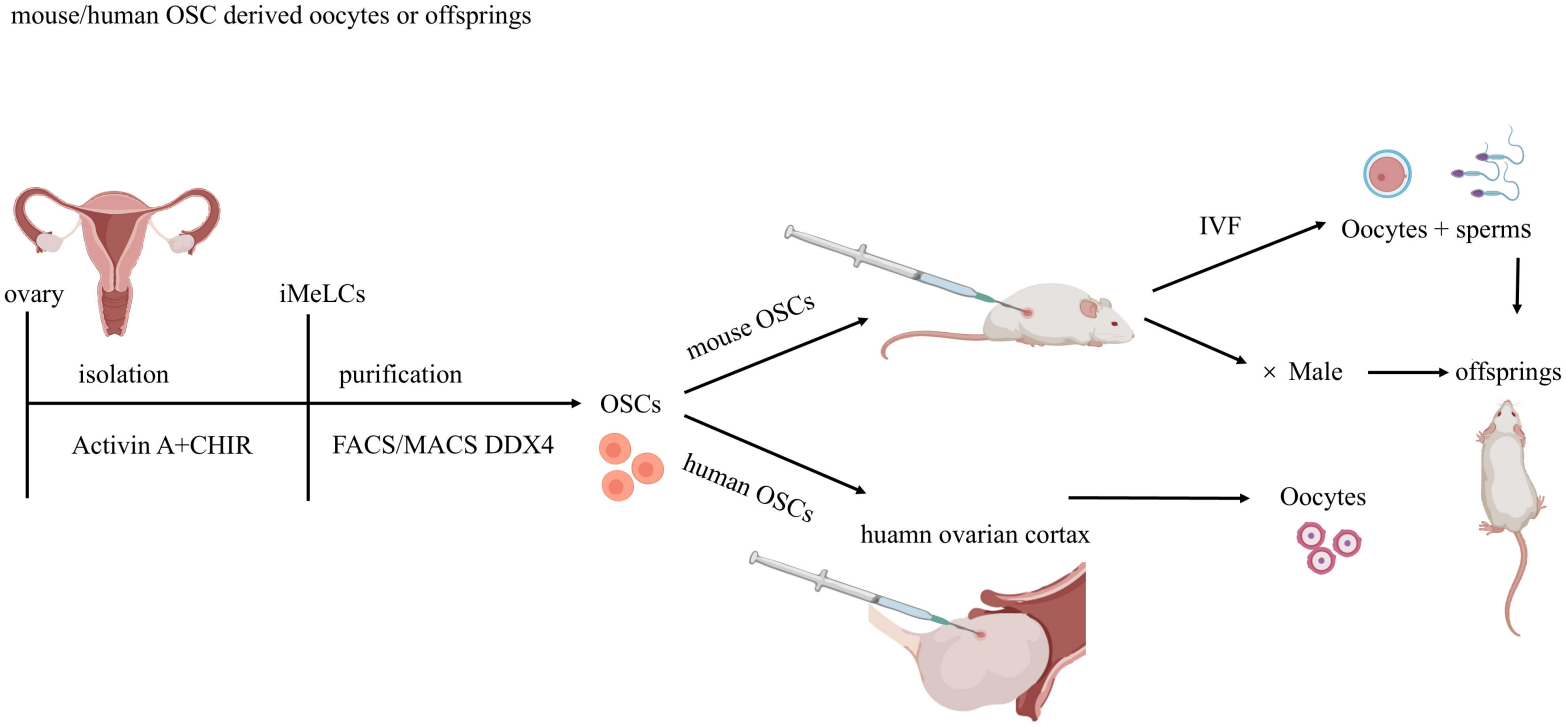
Methodologies for Ovarian Stem Cell Isolation, Purification, and Transplantation. Ovarian stem cells (OSCs) are isolated from murine /human ovaries using DDX4 antibody-based sorting via magnetic-activated cell sorting (MACS) or fluorescence-activated cell sorting (FACS).Mouse OSCs injected into recipient mouse ovaries undergo complete oogenesis, yielding functional oocytes that produce viable embryos and offspring post-fertilization. Human OSCs are injected into human ovarian cortex fragments and xenotransplanted into immunodeficient mice for further development.

Traditional views hold that most female mammals lose the ability to produce oocytes at birth and only have a limited reserve of oocytes. However, in 2004, Johnson et al. (28) reported the presence of reproductive cells with mitotic activity in the ovaries of both juvenile and adult mice, indicating their ability to continuously update the follicle pool. These mitotically active germ cells are referred to as OSCs [also known as female germline stem cells (FGSCs) or oogonial stem cells]. Subsequent studies provided further evidence of OSCs in mice and humans (4, 9, 12–16), and these cells were fertilizable and capable of generating embryos in murine models. Furthermore, hOSCs injected into human ovarian cortex tissue and then transplanted into mice, resulted in the cultivation of primordial follicles. Therefore, FGSCs have significant value in basic research and may provide new strategies for treating ovarian dysfunction and infertility, as well as delaying female aging (**Figure 4**; **Table 2**).

**Table 2:** Comparative methodologies for osc isolation, culture and transplantation.

Reference	Species	Source	Method	Antibodies	OSC culture	Transplantation
[4]	Mouse, Human	Human: reproductive age women (22-33years old)	MACS or FACS	C-terminal DDX4	**feeder:** mitotically-inactivated immortalized MEFs **OSC culture:** As described in Reference [13]	**Intraovarian injection of mouse OSCs:** GFP-expressing mouse OSCs (1 × 10^4^) were injected directly into each ovary of wild-type mice at 2 months of age. Between 7–8 months of age, transplanted mice were subjected to an induced ovulation protocol. **Intraovarian injection of human OSC and xenografting:** Human ovarian cortical tissue injected with approximately 1.3 × 10^3^ GFP-expressing human OSCs were grafted into NOD-SCID female mice
[13]	Mouse	5-day-old or adult C_57_BL/6×CD-1 F1 hybrid mice	MACS	MVH	**Feeder: **STO cell (derived from mouse SIM embryonic fibroblasts, strain SIM). **FGSCs culture medium:** MEM-α, 10% FBS, 1 mM sodium pyruvate, 1 mM NEAA, 2 mM L−glutamine, 0.1 mM β-ME, 10 ng/ml LIF, 20 μg/ml transferrin, 5 μg/ml insulin, 60 μM putrescine, 10 ng/ml mouse EGF, 40 ng/ml human GDNF, 1 ng/ml human bFGF and 15 mg/l penicillin.	**FGSC transplantation:** GFP-transduced FGSCs were transplanted into the ovaries of infertile mice, yielding offspring through natural mating with wild-type C_57_BL/6 males
[14]	Mouse	21-day-old Ddx4Cre; mT/mG mice	FACS	EGFP	**feeder: **SIM-6-thiogunanie-oualiain (STO) cell line. **FGSCs culture medium:** MEM alpha; 10% FBS, 30 mg/ml pyruvate, 2 mM L-glutamine, 50 mM β-ME, 6 mg/ml penicillin, 1 mM NEAA, 20 ng/ml mouse EGF, 10 ng/ml mouse bFGF, 10 ng/ml mouse GDNF, and 10 ng/ml mouse LIF	**FGSC transplantation:** FGSCs were injected into each ovary of infertile C_57_BL/6 mice.
[16]	Mouse, Human	**Mouse:** younger adult mice (6–8 weeks of age) **Human:** ovarian cortical tissue pieces	FACS	C-terminal DDX4	**Feeder:** mitotically inactivated mouse embryonic fibroblasts (MEFs) **OSC culture medium:** MEM-α, 10% (vol/vol) FBS, 1 mM sodium pyruvate, 1 mM NEAA, 1× Penicillin-streptomycin-glutamine solution, 0.1 mM β-ME, 1× N-2 supplement, 10^3^ units/ml LIF, 10 ng/ml EGF, 1 ng/ml bFGF and 40 ng/ml GDNF	**Intraovarian injection of mouse OSCs: FACS-purified GFP-expressing** mouse OSCs were injected into ovaries **Intraovarian injection of human OSC and xenografting:** Human ovarian cortical tissue injected with FACS-purified GFP-expressing human OSCs were grafted into NOD-SCID female mice
[18]	Mouse	day-old and adult mice	MACS	C-terminal DDX4	**Feeder:** STO cell **FGSCs culture medium:** MEM-α;1 mM NEAA,10 ng/ml mouse LIF, 2 mM L-glutamine, 1 mM sodium pyruvate, 0.1 mM β-ME, 10 ng/ml mouse LIF, 10 ng/ml mouse EGF, 40 ng/ml human GDNF, 1 ng/ml human bFGF, 10% FBS, and 15 mg/ml penicillin	**FGSC transplantation:** FGSCs were injected into each ovary of infertile mice.
[19]	Human	Ovarian cortical tissues from biopsies (srFGSCs) or follicular aspirates (faFGSCs)	DDX4	MACS	**Feeder:** STO cell **OSC culture medium:** MEMα, 10% FBS, 1 mM sodium pyruvate, 1 mM NEAA, 2 mM L-glutamine, 0.1 mM β-ME, 10 ng/mL human LIF, 10 ng/mL human EGF, 40 ng/mL human GDNF, 10 ng/mL human bFGF, and 6 mg/L penicillin	**Intraovarian EGPF^+^ faFGSCs injection and xenografting:** As described in Reference [4] **GV oocytes generation:** Feeder: GC monolayer ①basic medium containing 2μM RA (3 days) ②basic medium containing basic medium containing 10 ng/mL human EGF, 5μg/mL transferrin, 10μg/mL insulin, and 1 U/mL hCG and 1 U/mL PMSG(6 days) ③ basic medium containing basic medium containing 10 ng/mL human EGF, 5μg/mL transferrin, 10μg/mL insulin, and 1 U/mL hCG and 1 U/mL PMSG,1 ng/mL progestogen, 1 ng/mL 17-β -estradiol (E2), and 10% human follicular fluid (HFF) (3–6 days) basic medium: MEM-α, 15% FBS, 2 mM L-glutamine, 1 mM NEAA, 10 ng/mL human bFGF, 1 mM sodium pyruvate, 0.1 mM β -ME, and 6 mg/L penicillin
[20]	Mouse	6-or 16-week-old transgenic Pou5f1-EGFP mice	MACS	DdX4	**Feeder:** STO cell **FGSCs culture medium:** α-MEM, 10% FBS, 10 ng/ml mouse LIF, 10 ng/ml mouse bFGF, 10 ng/ml mouse EGF, 40 ng/ml mouse GDNF, 1 mM NEAA , 2 mM L-glutamine, 10 mg/ml penicillin, 30 mg/ml pyruvate, and β-ME	**3D ovarian organoid generation and culture:** FGSCs and female gonadal (from E12.5 to prenatal) somatic cells were mixed with Matrigel and cultured in 3D culture medium for 2 days. The co-cultures were transferred to transwell-COL membrane inserts, and and maintained in α-MEM-based medium for 4 days and followed by culture in StemPro-34-based medium
[40]	Mouse	5-day-old CD1 mice	MACS	Ddx4 or Fragilis	**Feeder:** STO cell **FGSCs culture medium:** As described in Reference [13]	—

bEGF, basic fibroblast growth factor; β-ME, β-mercaptoethanol; DDX4/Ddx4, DEAD box polypeptide 4; EGF, epidermal growth factor; EGFP, enhanced green fluorescent protein; FACS, fluorescence-activated cell sorting; FGSCs, female germline stem cells; Fragilis/ IFITM3, interferon-induced transmembrane protein 3; GDNF, glial cell line-derived neurotrophic factor; hCG, human chorionic gonadotropin; LIF, leukemia inhibitory factor; MVH:mouse vasa homologue protein; MACS, immunomagnetic bead sorting; NEAA, non-essential amino acids; OSCs, ovarian stem cells; PMSG, pregnant mare’s serum gonadotropin.

3D culture medium: GMEM; 15% KSR, 1.5μM retinoic acid, 2 mM L-glutamine, 1 mM NEAA, 30 mg/ml pyruvate, 100 mMβ-ME, 20 ng/mL mEGF, 10ng/mL bFGF, 10 ng/mL mouse GDNF, and 10 ng/ml mouse LIF, 30 mg/ml penicillin, and 75 mg/ml streptomycin.

α-MEM-based medium: α-MEM; 2% FBS, 2 mM L-glutamine, 100–300μM ascorbic acid, 20 ng/ ml mEGF, 50 mM β-ME, 30 mg/ml penicillin and 75 mg/ml streptomycin.

StemPro-34-based medium: StemPro-34 SFM; 10% FBS, 2 mM L-glutamine, 100–300μM ascorbic acid, 20 ng/ml mEGF, 50 mM β-ME, 30 mg/ml penicillin, and 75 mg/ml streptomycin, 800 nM ICI182780.

Whether or not OSCs exist in the adult mammalian ovary has been the subject of much debate. About 10 primary research papers questioned the existence of OSCs and/or postnatal oogenesis in mammals (32–37). A study rooted in single-cell RNA sequence analysis (scRNA-seq) of adult human ovarian cortical tissue claimed that OSCs do not exist and that other groups mistakenly identified perivascular cells (PVCs) as germ cells after isolating them via magnetic-assisted or fluorescence-activated cell sorting (38). However, follow-up studies have shown that human PVCs and germ cells separate into distinct clusters in scRNA-seq due to their non-overlapping gene expression profiles, preventing the misidentification of PVCs as OSCs in functional characterization studies (39). Other research groups mistakenly used PVCs, rather than germ cells, to isolate the original germ lineage cells using DDX4 antibody-based sorting. There may be several reasons for this: Firstly, OSCs are extremely rare in adult ovaries and can be difficult to identify. Secondly, due to improper workflow, the high degree of cell damage and death leads to non-specific antibody binding in FACS. Finally, the intrinsic autofluorescence of PVCs generates false-positive signals, resulting in their enrichment (39).

### 6.1 Isolation and culture of OSCs

#### 6.1.1 OSC isolation and purification

To isolate OSCs, a two-step enzymatic digestion method employing collagenase and trypsin is often used for the efficient digestion of ovary tissue (13). Subsequently, MACS or FACS techniques are used to purify and separate cells.

Both FACS and MACS rely on antibody-based detection of externally exposed protein epitopes on OSCs. In contrast to MACS, FACS yields significantly higher cell viability and purity. Notably, MACS remains widely adopted for isolating ovarian germ stem cells from heterogeneous populations. This technique combines magnetic beads with antibodies targeting specific membrane antigens. Multiple studies have successfully isolated OSCs using this approach (13, 15, 17, 19, 40), though potential contamination with non-target cells should be considered. Marker analysis of the DDX4-positive viable cell fraction obtained by MACS revealed several oocyte-specific mRNAs (4). This most likely reflects either a non-specific physical carry-over of small oocytes with OSCs during column washing and flushing or reactivity of cytoplasmic DDX4 in plasma membrane-compromised (damaged) oocytes with the antibody recognizing C-terminal DDX4. In addition, MACS also does not distinguish between viable and damaged or dead cells, and does not allow simultaneous assessment of other cellular features, such as yield, size or co-expression of additional markers. To overcome these limitations, White et al. explored using FACS for OSC isolation, building upon the MACS methodology (4). Dead-cell exclusion and cell size-based inclusion can be performed simultaneously using FACS, further enhancing the viability and purity of the cell population obtained. White and colleagues used an antibody directed against the COOH-terminus of Ddx4/DDX4 to purify rare mitotically active cells with gene expression profiles consistent with PGCs from adult mouse ovaries and human ovarian cortical tissues via FACS. In addition, unlike the oocyte contamination observed when OSCs were isolated by MACS using the antibody recognizing C-terminal DDX4, use of this antibody with FACS provides a superior strategy to obtain OSCs free of oocytes.

In addition to the validated and independently verified OSC purification method based on DDX4 antibodies, an antibody targeting the extracellular domain of interferon-induced transmembrane protein 3 (IFITM3/Fragilis), a marker for PGCs, can also be used with greater efficiency to isolate mOSCs (40). In recent years, OSCs have been isolated from mouse or human ovaries by MACS or FACS based on anti-DDX4 antibodies. However, Zhang and Wu explored a novel isolation method using endogenous fluorescent proteins rather than exogenous antibodies to isolate DDX4-positive cells from postnatal ovaries of lineage-tracing mice (Ddx4-Cre;mT/mG mice) (14). Subsequent experiments demonstrated that these isolated cells could establish cell lines capable of generating offspring.

#### 6.1.2 OSC culture

Mouse and human OSCs are often established on mitotically inactivated STO feeder cells [derived from Sandos inbred mice (SIM) 6-thioguanine-resistant, ouabain-resistant embryonic fibroblasts]. Although an underlying monolayer of somatic feeder cells is not absolutely required for the successful establishment of mouse or human OSCs *in vitro*, the initial rate of proliferation is greatly enhanced using a co-culture system (16). The culture medium for OSCs usually consists of MEM-α, FBS, sodium pyruvate, non-essential amino acids (NEAA), L-glutamine, β-mercaptoethanol (β-ME), LIF, EGF, glial cell line-derived neurotrophic factor (GDNF), basic FGF and penicillin (4, 13, 18, 20). Some media also incorporate transferrin and insulin (4, 13).

### 6.2 Characterization of OSCs

As germline stem cells, OSCs not only maintain oogenesis, but also have the characteristics of adult stem cells. The proportion of OSCs is very small, accounting for about 0.014% ± 0.002% of cells in mouse ovaries (4). The main characteristics of mouse and human OSCs are summarized as follows. 1) OSCs are similar to spermatogonial stem cells (SSCs) in morphology, with a larger nucleus and less cytoplasm. 2) Most OSCs are positive for PRDM1/BLIMP1, DPPA3/STELLA, IFITM3/Fragilis, DAZL, OCT4, REX1/ZFP42 and TERT. However, OSCs do not express oocyte specific markers, such as newborn ovary homeobox (NOBOX), zona pellucida glycoprotein 1–3 (ZP1–3), growth differentiation factor 9 (GDF9), NANOG, SSEA1 or SOX2, c-KIT, FIGLA, and synaptonemal complex protein 1–3 (SCP1–3). Oocyte contamination can be excluded by examining oocyte-specific markers. 3) FGSCs show high telomerase activity and most of them have a normal karyotype. 4) FGSCs are also positive for alkaline phosphatase staining. 5) OSCs show a female imprinting pattern. Differentially methylated regions in two maternally imprinted regions (Igf2r and Peg10 regions) and two paternally imprinted regions (H19 and Rasgrf1 regions) were observed, indicating that the maternally imprinted regions were partially methylated and the paternally imprinted regions were demethylated in OSCs.

### 6.3 Oocytes derived from OSCs

As mentioned earlier, the generation of oocytes from OSCs typically necessitates an *in vivo* environment. mOSCs can be microinjected into the ovaries of recipient mice. In contrast, hOSCs need to be injected into human ovarian cortex tissue first, and then xenotransplanted into immunodeficient mice to achieve development.

#### 6.3.1 Intraovarian injection of mouse OSCs and generation of offspring

Multiple independent studies have confirmed that mouse OSCs can differentiate into functional oocytes *in vivo*, yielding viable embryos and offspring. To identify oocytes derived from OSCs, OSCs are often transformed to express fluorescent reporter genes, such as GFP. After stable integration of GFP, GFP-expressing mOSCs can be directly microinjected into the ovaries of infertile female mice pre-treated with cyclophosphamide and busulfan for further development. Determination of oocyte generation includes the following three approaches. First, histological evaluation of ovaries injected with OSCs to observe GFP-positive oocytes. Second, induction of ovulation using gonadotropins, followed by *in vitro* fertilization of these oocytes with wild-type sperm and monitoring embryo development. Third, mating trials were conducted by pairing recipient female mice receiving intraovarian GFP-OSC injections with wild-type male mice. Conventional PCR-based genotyping is then performed to determine whether the oocyte fertilized *in vivo* to produce a given offspring was derived from the recipient (wild-type) or from the transplanted OSCs (GFP-positive).

In addition to transplanting OSCs back into ovaries, *in vitro* methods for generating oocytes from mOSCs have also been explored (4). These include the *in vitro* culture of OSCs with ovarian somatic cells to form organoids (20). Within these organoids, OSCs gradually develop into follicles and after 3D culture, these follicles mature into complexes containing immature oocytes, known as ovarian follicle-oocyte complexes. Mature oocytes can then be successfully obtained using *in vitro* maturation techniques, *in vitro* fertilized, and the embryos transplanted. The success rate of the *in vitro* fertilization was not significantly different from that of the control group (49.9% and 46.2%, respectively). Offspring born from the transplants exhibited normal weight, methylation patterns, and fertility.

#### 6.3.2 Injection of human OSCs into human ovarian cortical tissue and xenografting

mOSCs have been extensively researched, and *in vitro* and *in vivo* protocols have been developed for the induction of mOSCs to form oocytes or embryos that can produce offspring. However, few studies have examined oocyte formation from hOSCs. The conventional method is to inject hOSCs into human ovarian cortical tissue and then transplant them into mouse ovaries (4) or subcutaneous sites (16, 19) for further development. This produces GFP-positive follicles in xenografted OSC tissues and shows that hOSCs, like mOSCs, have the potential to develop into oocytes and to supplement the follicular pool. hOSCs have recently been used to further explore the *in vitro* development of human oocytes.

It is worth mentioning that Ding (19) established FGSC lines from scarce ovarian cortical tissues that exist in follicular aspirates (faFGSCs), which are produced and discarded by *in vitro* fertilization centers around the world. A three-step differentiation protocol for differentiating oocytes from faFGSCs was developed. First, faFGSCs were cultured on a granulosa cell monolayer for 3 days in medium containing bFGF and retinoic acid (RA) and thereafter, for 6 days in the presence of bFGF, EGF, insulin, transferrin, PMSG (pregnant mare’s serum gonadotropin), and hCG (human chorionic gonadotropin). Subsequently, E2 (17-β-estradiol), progestogen, and human follicular fluid were added to the medium and culture continued for 3–6 days. Oocyte-like cells appeared continuously during the differentiation process, and some oocyte-like cells (4.9%) were observed to have germinal vesicle-like structures. These findings indicate that the rare ovarian tissue in follicular aspirates can be used as an alternative source of FGSCs, which is a promising solution for the shortage of adult human ovarian cortex tissue. Furthermore, human germinal vesicle oocytes could be developed *in vitro* from faFGSCs under defined culture conditions.

#### 6.3.3 Analysis of *in vitro*-derived oocytes

The expression of oocyte-specific markers in oocytes derived from human and mOSCs is routinely analyzed using PCR or immunofluorescence cytochemistry to confirm their cellular identity. Oocytes derived from mouse and human OSCs typically express key markers such as DDX4, KIT, YBX2 (also known as MSY2 and as CONTRIN in humans), NOBOX, LHX8, GDF9, ZP1, ZP2, and ZP3.

## 7 Conclusion and perspectives

In the last decade, rising infertility rates in developed countries of 12%–24% have increased efforts to establish *in vitro* conditions for self-renewal and differentiation of PGCs. Significant progress has been made in producing oocytes *in vitro* using PSCs. The successful generation of oogonia-like cells from hPSCs offers a new possibility for human *in vitro* gametogenesis. The discovery of OSCs has opened up new areas of research in human reproductive biology, which will address infertility issues and women’s reproductive health. This will provide women with more choices. For example, OSCs can be cryopreserved for future treatment of infertility. Moreover, purified human OCS can act on the ovaries themselves, increasing the reserve capacity of ovarian function.

Although mOSC-derived oocyte development has been extensively studied, further exploration is needed to apply findings in mice to humans. First, unlike the “naïve” state characteristic of mouse ESCs and iPSCs, hESCs and hiPSCs exhibit a “primed” state and exist as heterogeneous populations with varied differentiation potential. Second, the *in vivo* gonadal environment is essential for meiosis and, to generate functional oocytes from PSCs, the PSCs need to be transplanted into an ovary, which is restricted by ethical and safety considerations.

In addition, the internal gonadal environment is necessary for meiosis. PGCLCs obviously need to interact with fetal gonadal somatic cells to differentiate into mature oocytes (1, 2). Studies in mice have shown that PGCLCs introduced in adult ovarian tissue generate only immature oocytes that arrest and degenerate at very early stages of follicle development. Therefore, obtaining human fetal gonadal tissue and making it available for any type of clinical platform involving germ cell development from PSCs remains to be addressed. In contrast, OSCs are capable of generating mature oocytes when introduced into adult ovarian tissue, removing the need for fetal ovarian somatic cells. However, OSCs alone are not capable of differentiation into fully functional eggs. OSCs, like all other stem cells, require parallel incorporation of appropriate somatic cell partners or transplantation *in vivo* to be successful. Without question, the most important of these partners are primitive or undifferentiated granulosa cells (sometimes referred to as pregranulosa cells), which are capable of interacting with newly generated oocytes to form primordial follicle-like structures. A new method to differentiate hiPSCs into granulosa-like cells may offer more possibilities for oocyte development (41).

Finally, the specification of PGCLCs from ESCs or iPSCs may fail to account for the importance of the germline mitochondrial DNA (mtDNA) bottleneck in ensuring maternal passage of ‘clean’ mtDNA generation after generation (42). Even in mice, it remains to be determined, if nuclear reprogramming of differentiated somatic cells into iPSCs for subsequent generation of PGCLCs generates germline cells that effectively carry out the process of maternal mitochondrial inheritance. In other words, studies must be performed to demonstrate that offspring produced from iPSC-derived eggs are not burdened from the outset with compromised mitochondrial genomes (43). In comparison, OSCs show some advantages in this respect. Unlike ESCs and iPSCs, OSCs are unipotent and programed from the start as a germ lineage; therefore, these cells require no directed differentiation or genetic manipulation to achieve a germline identity that is capable of oogenesis (43).

In addition, the source of OSCs needs to be resolved. Research on hOSCs is limited by the difficulty of obtaining ovarian tissue and related ethical issues. The previously mentioned faFGSCs (19) can serve as an alternative source of OSCs. Furthermore, studies in mice suggest that bone marrow and peripheral blood may be a potential source of germ cells (44, 45). Bone marrow or peripheral blood transplantation were performed on female mice sterilized by chemotherapy. After transplantation, donor-derived oocytes were produced in the recipient’s ovaries. Their morphology, enclosure within follicles, and expression of germ cell- and oocyte-specific markers collectively support these cells as *bona fide* oocytes. However, mating experiments revealed that all offspring were derived from the recipient germline. Donor-derived oocytes were only observed in immature follicles up to the preantral stage of development but never observed in mature antral or Graafian follicles from which ovulated eggs are derived (45). These findings indicate that bone marrow transplantation and peripheral blood transplantation can improve follicular development and fertility, but that bone marrow stem cell-derived oocytes do not contribute to the pool of ovulated eggs used for reproduction (43).
